# Implementing evidence-based care for paediatric obsessive-compulsive disorder in public mental health services: clinician perspectives of barriers and enablers

**DOI:** 10.1080/00049530.2026.2692599

**Published:** 2026-07-29

**Authors:** K. M. Dyason, S. Wilson, P. Pham, L. J. Farrell, J. R. Grisham, N. Taylor, B. Tapp, T. Dyson, B. Richmond, T. Bustos, K. Knight, C. Gaskin, I. E. Perkes

**Affiliations:** a Discipline of Psychiatry, University of New South Wales, Sydney, Australia; b Department of Psychological Medicine, Sydney Children’s Hospitals Network, Sydney, Australia; c Child and Adolescent Mental Health Services, South-Eastern Sydney Local Health District, NSW Health, Sydney, Australia; d Discipline of Paediatrics, University of New South Wales, Sydney, Australia; e Griffith Centre for Mental Health and School of Applied Psychology, Griffith University, Southport, Australia; f School of Psychology, University of New South Wales, Sydney, Australia; g Implementation to Impact (i2i), School of Population Health, University of New South Wales, Sydney, Australia; h School of Psychology, University of Sydney, Sydney, Australia

**Keywords:** Obsessive-compulsive disorder, implementation, public health, child and adolescent mental health services, type 2 trial, child

## Abstract

**Objective:**

To evaluate the feasibility and acceptability of a model of care for paediatric obsessive-compulsive disorder (OCD), by outlining the barriers and enablers experienced by clinicians implementing the model in routine public health settings.

**Method:**

Six child and adolescent public mental health services participated in a Type 2 implementation and effectiveness trial of an OCD model of care. Clinicians were trained in screening, assessment, psychoeducation and group-based exposure with response prevention. Focus group interviews were conducted at the conclusion of the trial with clinicians from each service. Interview responses were guided by a logic model to elucidate enablers and barriers to implementation, considering resources, moderators, mediators and outcomes.

**Results:**

Implementation varied across services. Three key themes emerged. Firstly, existing resources were directly and inversely linked to implementation. Secondly, OCD training and specialised clinical support increased clinicians’ knowledge and skills, benefitting both clinicians and clients. Finally, case complexity influenced how and when implementation occurred.

**Conclusions:**

The model of care was feasible and acceptable. Adjuncts to clinical service delivery (e.g. attending training) were easier to implement than changes to existing procedures (e.g. introducing screening). Aligned with the UK NICE guidelines, this model of care may be suitable for public Australian services to improve OCD detection and treatment for young people.

## KEY POINTS

What is already known about this topic:

(1) Despite known effective evidence-based treatments for paediatric OCD, there remain substantial treatment and implementation gaps, meaning many children do not receive adequately dosed and high-fidelity treatment.

(2) The United Kingdom has had extensive treatment guidelines for OCD since 2005, through the NICE guideline publications.

(3) However, Australia does not have any service plans or clinical guidelines for OCD, resulting in clinical variation, reduced dose treatment and continued “treatment and implementation gaps”. Prior to the current study, there were also no public speciality services for paediatric OCD.

What this topic adds:

(1) Implementing a tiered model of care within public child and adolescent mental health services and a public children’s hospital was feasible and acceptable to clinicians and service managers.

(2) Implementation of recommended evidence-based screening, assessment and treatment was moderated by existing resourcing (including staffing and administrative supports), clinical complexity and ability to communicate clearly across and within teams.

(3) Adjuncts to clinical service delivery (e.g. attending training) were easier to implement than changes to existing procedures (e.g. introducing screening).

Obsessive-compulsive disorder (OCD) affects 2–3% of children and adolescents (Geller, 2006) with a typically chronic, fluctuating course (Geller et al., 2021; Piacentini et al., 2003; Pinto et al., 2006) persisting across the lifespan (Stewart et al., 2004). In Australia, that’s approximately 47,000 children aged 7–17 years living with diagnosable OCD (based on population estimates [Australian Bureau of Statistics, 2025]). Despite effective treatment options (McGuire et al., 2015; NICE, 2005a; Öst et al., 2016; Skapinakis et al., 2016), OCD has an unacceptably long duration of untreated illness, noted both internationally (Albert et al., 2019; Dell’osso et al., 2019; Fineberg et al., 2019) and in Australia (Cooper et al., 2023) – second only to bipolar affective disorder, type 1, among psychiatric conditions (Altamura et al., 2010). Early intervention is key: a longer duration of untreated illness is associated with poorer response to treatment (Perris et al., 2023) and delayed developmental milestones and identity formation in children and adolescents (Fineberg et al., 2019). A “treatment gap” exists where many children and adolescents do not access evidence-based treatments due to lack of detection and poor access to treatment (Farrell et al., 2023; Senter et al., 2021). Further, when they do access treatment, there is an “implementation gap”: the first-line recommended treatment of exposure with response prevention (ERP) is not provided to a high fidelity or a full “dose”, or is not provided at all (Farrell et al., 2023).

To respond to these challenges, the UK created NICE guidelines for OCD and body dysmorphic disorder (BDD) in 2005, which were implemented nationally for a coordinated approach to assessing and treating OCD and BDD in the public health system (Lovell & Bee, 2008). The guidelines outline evidence-based recommendations for assessment and treatment, organised into three phases and six stepped-care treatment options. The three phases are: (1) awareness and recognition, involving specialist multi-disciplinary teams to increase awareness and disseminate evidence-based knowledge to clinicians and the general public; (2) recognition and assessment of OCD in healthcare; and (3) stepped treatment options once accurately diagnosed, with less intense treatment (e.g., self-help and brief or group cognitive-behavioural therapy) recommended for mild OCD and more intense treatment (e.g., intensive individual or inpatient treatment) recommended for severe or refractory OCD. Specialist multidisciplinary teams provide training and knowledge dissemination, while national organisations (e.g., OCD UK) raise awareness with the general public as well as clinicians. National screening for OCD in healthcare settings is strongly recommended, with the rationale that OCD is relatively prevalent across the lifespan, treatable, and often secretive and not disclosed without direct questioning (Fineberg et al., 2008; National Institute for Health and Care Excellence [NICE], 2005a). The public health system (NHS) delivers treatment free of charge, covering lower-intensity 10–20 session ERP treatment to higher-intensity inpatient treatment, including at specialist OCD centres (OCD UK, 2018). The NHS has invested in systematic reviews on the effectiveness and cost-effectiveness of psychological treatments (e.g., Skapinakis et al., 2016), and NICE has freely available implementation supports online, such as costing tools, audit criteria and links to treatment manuals (National Institute for Health and Care Excellence [NICE], 2005b).

Whilst Australia has a different healthcare system to the UK, including higher availability of private sector services, the public health system is relatively similar. Yet in Australia, there are no service plans or clinical guidelines for OCD, and prior to the creation of the model of care (MoC) described in this paper, there were no publicly available paediatric specialist OCD services. Psychological treatment is largely provided by the private system, of which up to 10 individual and 10 group sessions per year are subsidised by the government through the Better Access initiative (Australian Department of Health, Disability and Aging, 2025).

Public mental health services for children and adolescents are available through Child and Adolescent Mental Health Services (CAMHS) for moderate-severe mental health conditions. Brief early intervention services are available through the organisation headspace for young people aged 12–25 years, which is partly funded by the state/local health services and partly through the Better Access scheme. The brevity of sessions and the expansive, multi-purpose nature of services without national guidelines result in clinical variation, reduced dose treatment and continued “treatment and implementation gaps”.

To address these gaps, a nationally consistent framework is needed to equip clinicians in health services to screen and assess for OCD and effectively treat OCD across the lifespan and spectrum of severity – from lower to higher intensity treatments, up to and including hospital inpatient admissions. Given the similarities in public health services, the NICE guidelines made a logical and appropriate choice to adapt for an Australian paediatric public health setting. We therefore developed and implemented a tiered MoC aligned with the NICE guidelines and based on research findings, clinical expertise and consultation with clinicians and consumers (Perkes et al., 2022) for children and adolescents. Similar to the UK’s model, we developed a specialist service (akin to phase 1 in the NICE guidelines), focused on screening and assessment (akin to phase 2) and provided tiered treatment options based on these detailed assessments (adapted phase 3).

The MoC (Perkes et al., 2022) comprises: (1) OCD screening for all clients presenting for clinical services in CAMHS/headspace; (2) for those screening positively for OCD, conducting a thorough assessment of OCD for diagnosis and to inform treatment selection; (3) for clients with mild-moderate OCD, offering group ERP therapy (Farrell et al., 2012; Lavell et al., 2016); and (4) as an alternative to inpatient hospital admission for clients with severe-extreme OCD, offering intensive, individual ERP (Farrell et al., 2022) in the community. Other wrap-around care was also provided in the MoC by a speciality team but not detailed here, such as consultant-liaison services, medication reviews, an online clinician directory of private providers with expertise in OCD (Cooper et al., 2023), and monthly continuing professional development forums/seminars open nationally. This paper describes the experiences of clinicians and health service managers from teams who implemented the MoC to determine feasibility and acceptability and to understand the barriers and facilitators to implementation within existing public healthcare settings. We have evaluated other aspects of the MoC in a series of papers: screening implementation (Dyason, Pham, et al., 2025), clinician training outcomes (Racz et al., 2025), intensive ERP effectiveness (Dyason, Perkes, et al., 2026) and the impact of implementation on inpatient hospitalisation (McAdams et al., 2026).

## Method

This effectiveness and feasibility implementation trial was pre-registered with ethics approval (Sydney Children’s Hospitals Network, 2022/ETH01213; Griffith University, 2022/756).

### Implementation setting

A newly established multidisciplinary OCD specialty team in a children’s hospital in Sydney, Australia, partnered with a geographically co-located local health district to implement a MoC within all mental health services in the district for 0–17 years, including CAMHS and affiliated services (e.g., Headspace). CAMHS offer multi-disciplinary mental health services to children and adolescents up to 18 years with moderate to severe emotional, behavioural and social difficulties requiring short- to medium-term assistance (NSW Health, 2021). Headspace provides brief, early intervention to young people aged 12–24 years for subclinical and mild mental health conditions (McGorry et al., 2007). Six teams piloted the MoC for 12 months: four CAMHS services, one headspace centre and one specialist team for youth at risk of psychosis. The local health district covers an area of 468 square kilometres and includes 167,377 children aged 0–17 years (NSW Department of Planning and Environment [NSW], 2022). Using a 2% prevalence rate and excluding the 0–4 year age group, there are an estimated 2394 children per year with OCD in the catchment. CAMHS and the at‑risk‑of‑psychosis teams in the local health district each see between 250–400 children and adolescents per year; information was not available on headspace caseload numbers. The OCD team offered training and implementation support in addition to providing some direct assessment and treatment for severe OCD.

*Training*. At least two clinicians from each CAMHS/affiliated service completed a 2-day workshop orienting them to the MoC and providing training in the fundamentals of evidence-based OCD screening, assessment and group-based ERP using the evidence-based OCD Busters program (Farrell et al., 2012; Lavell et al., 2016). Clinician knowledge and competency were assessed through a variety of methods, including role plays, detailed elsewhere (Racz et al., 2025). Six supervision sessions with an experienced clinical psychologist and expert in ERP (LJF) were provided to clinicians facilitating treatment groups during the implementation period. Further in-service training sessions were held, open to all clinicians at participating services, focusing on MoC implementation and basic training in OCD screening, assessment and psychoeducation. Drop-in implementation and assessment support sessions were held weekly by the OCD team (KMD, clinical psychologist, and SW, clinical nurse consultant), open to clinicians, team leaders or administrators from participating services. Project oversight, including clinical and leadership governance, was provided by a quarterly steering committee comprised of hospital leaders, and monthly working groups comprised of key clinicians and team leaders from each service.

### Participants

All team members from all six services were invited to participate in qualitative interviews to evaluate implementation, including those who did not complete the 2-day training workshop. A total of 19 team members from 5 services participated in the interviews: 10 clinicians who had attended the 2-day training workshop, 3 clinicians who had not, 2 team leaders/managers who attended the training and 4 who had not. No administrators attended interviews. Between two to six team members per team attended the interviews, representing feedback on behalf of the whole team. Some additional team members who could not attend wrote feedback to submit to the interviewers. Team members came from a variety of professional backgrounds, e.g., psychologists, social workers and nurses. One team declined to participate in the interviews due to team disruption while the service relocated to another building.

### Qualitative interview procedure

After the 12-month implementation, all six services were invited to 1-hour in-person qualitative feedback interviews regarding the implementation process, conducted by two members of the OCD team (SW, clinical nurse consultant, BR, provisional psychologist, KMD, clinical psychologist, IEP, child and adolescent psychiatrist) in August and September 2023. Interviewees were advised that their aggregated and deidentified data may be used for research prior to completing the interviews and could opt out of participating in the interview; as such, they provided assumed consent. Staff were not financially compensated for either implementing the MoC or participating in the qualitative interviews, as implementing the MoC was part of their employment, as the local health district adopted the MoC as its model for assessing and treating OCD across all services.

A blank copy of the logic model framework (Mills et al., 2019; see Supplementary Material) was projected on a whiteboard or printed on paper to guide responses. To gather information on participants’ experiences, each “activity” within the MoC (e.g., screening, group ERP, intensive program and training) was assessed against the resources necessary to complete each activity, factors moderating how the MoC was implemented and mediators between initial (short-term) to sustained (long-term; hypothetical) implementation. Moderators were separated into inner factors, such as individual clinician characteristics, organisational support, policy fit and service pressures, and outer factors, such as team set-up, leadership, relationships between teams and staff experience, skills and interest (Mills et al., 2019). Mediators were separated into short-term outcomes, such as establishing systems, shared agendas, client experiences and training, and long-term outcomes such as an embedded MoC and sustained improvements to client care (Mills et al., 2019).

Questions stems (Supplemental Material) were used to generate content for each of the cells in the model. All interviews used the same question stems; the stems were not iteratively refined across interviews. Comments from interviewees were transcribed live onto the electronic whiteboard or a large piece of paper by a member of the interviewing team, visible to all interviewees in the room, permitting immediate clarification and consensus, even though verbatim wording was not transcribed nor audio recorded, and comments were not assigned to individual interviewees. The completed logic model table for each service was emailed to interviewees from that service, providing further opportunity for feedback.

After all interviews were completed, two composite logic models were generated from each team’s individual logic model table by three members of the OCD team (KMD, clinical psychologist, SW, clinical nurse consultant, BR provisional psychologist) using focused coding to combine similar or repeated themes across services, coding each as either a barrier or facilitator; no data were excluded from the composite logic model. Constructivist grounded theory (Charmaz, 2000) loosely guided the coding of the composite logic models through the initial co-construction of meaning between the research team and interviewees when representing interviewee’s comments in the table; in the iterative way that the final composite model was created, adding or refining themes to represent all comments across the interviews; and in distilling the themes in the composite models to the general themes identified in the Results. Disagreements were rare but resolved through discussion until consensus was reached.

## Results

The composite logic models outline the barriers (Table 1) and enablers (Table 2) to implementing the MoC, representing all comments across teams. Each activity in the MoC was evaluated against components of the Logic Model: resources, moderators (inner factors and outer factors) and mediators (short-term and long-term). Three themes emerged across the logic models impacting how services were able to implement the MoC: pre-existing structures, supports and resourcing; access to training and specialisation; and the impact of risk or clinical complexity.

**Table 1.** Barriers to implementing the components of the MoC as identified by six teams.

	MoC Elements (Activities)						
	Screening	Assessment	Group	Intensive	Training and Supervision	Monthly CPD	Integration of Model
Resources	*No existing screening protocols* *Staffing – Limited capacity to action screening* *Integration into existing clinical interview* *Prioritisation: co-morbid diagnosis, risk*	*Lack of capacity to assess and follow up* *Onward referral for assessment doesn’t fit clinical model* *lengthy referral process*	*Physical room space* *Long duration of program* *Group running out of hours* *Impact on other clinical responsibilities* *Time cost* *Insufficient training* *Not enough staff trained* *Age appropriateness of workbooks*	*Clinicians not trained*	*High staff turnover* *Lack of staff trained* *Time consuming* *Prioritisation of OCD training over other training* *Lack of training in screening, assessment, and diagnosis* *Unaware of resource/time cost upfront*	*Clinician time*	*Staff capacity* *OCD not prioritised* *Lack of trained staff* *Lack of access to resources across team* *Funding structures don’t allow for all components of Model*
Moderators: Inner Factors	*False positives* *Lack of specificity of screener* *Clinician competence to follow up* *De-prioritisation of clinical judgement of how/when to screen* *Paper/electronic screening* *Perceived burden on clients* *Appropriateness of screening at first assessment*	*Client motivation to complete assessment* *Parent/carer involvement* *Difference in reports from child/carer* *Time cost* *Different EMRs between services* *Need for more training* *Differential diagnosis not considered* *Unsure of process* *Lack of guidance interpretating measures* *Prioritisation of other needs e.g.*, *crisis*	*Cost benefit ratio of resources* *Few OCD clients* *Client comorbidities* *Capacity for family review sessions* *Capacity for out of session follow up* *High staff turnover* *Mismatch between session content and client progress* *Lack of consult before running group*	*Referral process unclear*		*Lack of awareness of CPD* *Limited CPD content ahead of time*	*Inadequate fit within service as unable to service 18–25yo* *Lack of clients with OCD* *No system for recording activity-based funding* *Complexity of clients*
Moderators: Outer factors	*Lack of guidance on procedures*		*No clinician manual* *Parent sessions hard to contain* *Lack of case management* *Communication between services* *Client suitability for group* *Low number of clients* *Lack of universal systems access* *Untrained clinicians unable to support clients with group content*		*Need all staff trained* *Lack of practical skills training* *Lack of ERP examples* *Research integration*		*Running groups across services – different documentation systems/handover process* *Ineffective communication* *Processes unclear* *Cost-benefit ratio* *Pressure to create group*
Mediators: Short-term outcomes		*Lack of suitable clients* *High staff turnover*	*Limited benefit to client* *Feasibility of concurrent treatments* *High dropout rate*	*Clients missing school*	*Lack of practical ERP skills*		*Families unclear on process*
Mediators: Long-term outcomes

**Table 2.** Enablers to implementing the components of the MoC as identified by six teams.

	MoC Elements (Activities)						
	Screening	Assessment	Group	Intensive	Training and Supervision	Monthly CPD	Integration of Model
Resources	*Pre-existing screening protocols* *OCD primary presenting problem*	*Specialist team available* *Easy referral process*	*EMR templates* *Dedicated planning time prior to group*		*Knowledge on follow up questions* *More staff trained*		
Moderators: Inner Factors	*Clinical judgement incorporated into screening* *Preference for verbal screening*	*More clinician training* *Telephone assessment* *Parent/carer involvement* *Involvement of child/young person* *More choice in assessment selection* *Use of clinical judgement for timing of assessment*	*Increased clinician confidence in ERP*	*Second opinion* *Good fit for CAMHS*	*1:1 supervision* *Drop-in sessions* *Staggered training*		*Intensive good fit for CAMHS* *More training* *Admin support*
Moderators: Outer factors		*Good communication between services* *Comprehensive outcome letter* *Recommendations*	*Clinician enjoyment* *Process for shared communication with other services* *Use of EMR notes to monitor client progress*	*Shared care* *New treatment* *Approach* *New case formulation*	*Refresher course* *Good overview of OCD* *Trainers’ experience and analogies* *Solid theoretical grounding* *Scheduled supervision* *Preparing for supervision in advance*	*Appropriate for complex cases* *Drop-in sessions*	*Face-to-face communication* *Supervision* *More staff trained*
Mediators: Short-term outcomes	*Raised awareness of OCD* *Raised awareness of need to screen* *Raised saliency of OCD*	*High detection rate* *Uncover previously undetected symptoms* *Facilitated conversations*	*Helpful parent sessions* *Benefit from group* *Peer support (client and parent)*	*Clinicians learning through co-facilitation and observation*	*Increased confidence implementing ERP* *Quality training* *Training on how to screen* *Quality supervision* *Case consult* *Observation/* *co-facilitation of intensive*	*See benefit* *Useful guidance* *Up to date knowledge* *Evidence-based knowledge*	*Drop-in sessions* *Ability to co-facilitate/* *observe intensive*
Mediators: Long-term outcomes	*Development of digital screener* *Opt-in screening* *Verbal screening*		*Professional development* *Better staff retention*	*Positive outcomes* *Scheduling* *Maintenance sessions*	*Future training for 1:1 ERP*		*Consultation available* *Increased awareness of OCD* *Highlighted need for OCD services* *Trained clinicians* *Helpful supervision* *Professional development* *Communication with specialist team* *Enjoyment*

### Theme 1: existing structures, supports and resourcing

The most common barriers and enablers relating to resourcing across various activities were often directly and inversely linked. For example, the two services already routinely using screening measures reported finding the screening measure easier to implement, whereas the remaining three services needing to generate de novo processes reported more difficulties and completed the measure less frequently (Dyason, Pham, et al., 2025). Similarly, having existing administration supports for screening, making referrals or scheduling/organising treatment groups was an enabler to implementation; in services with less supports, this was an additional burden on clinician time and therefore a barrier to implementation. Some services commented that they were unaware of the time and resource cost of implementing the MoC before commencing implementation or did not sufficiently understand the research evaluation components of implementing the MoC (e.g., which elements of the MoC required active research consent vs a waiver of consent from clients to use their data for research purposes; whether clients of the services could access elements of the MoC without consenting to their data being used for research purposes).

Staffing was a common theme across services and activities. Almost all services reported workforce shortages, limiting their ability to release staff for training, dedicate time to activities outside of direct service provision or provide more in-depth clinical services as per the MoC, e.g., assessments or between-group contact for group participants. Respondents proposed that staggered training or refresher courses would enable more staff to be trained with less impact on clinical service delivery. Sustainability in relation to staff turnover was a concern to all services: specialised knowledge was lost from the service when clinicians resigned, and new staff needed to be trained. The 2-day workshop was a drawback for managers, as clinician time attending the workshops meant a reduction in service delivery and potentially a de-prioritisation of other clinically relevant training (e.g., family-based treatment for eating disorders). Indeed, the trade-off of resourcing and clinician time was often raised as both a barrier and an enabler. For example, while running groups hypothetically meant more clients could receive services with the same amount of clinician time, time costs to get a new initiative off the ground were substantial and did not immediately result in time saved. Allocated time to prepare for groups and supervision was seen as an enabler to implementation.

Physical space was a barrier to running groups for all services: rooms needed to be large enough to accommodate at least five clients and two clinicians, and for 3 of the 10 sessions a separate large room was required for accompanying parent sessions. Clients and clinicians sometimes needed to travel between sites to attend groups, which required more logistical organisation and time burden. Clinicians reported difficulties communicating with other services for shared care during the group program, especially where different electronic medical record systems were used, and/or they were unsure which service was responsible for out-of-session contact and sessions. To better fit the school timetable, two services ran groups outside of their standard operating hours, necessitating clinicians and administrators changing their working hours and personal schedules. The MoC was only funded for ages 7–17, yet two services also treated young people aged 18–25, creating inconsistency, frustration for staff and reducing the available service offerings. The age range of the MoC was therefore a substantial barrier in these two services.

Overall, comments were mixed regarding communication pathways, knowledge of referral processes and supports offered by the specialist team. Some participants noted that communication between the specialist service and their team was an enabler and they found the various support options (e.g., email, drop-in sessions, referrals and supervision) met their needs, that referral processes were clear and easy and that resources (e.g., note templates and group workbooks) were useful. Face-to-face communication was preferred by most participants. In contrast, other services commented that they were unsure of referral processes, found referral processes lengthy, did not know how to contact the specialist team and/or were generally unaware of the processes or services offered.

### Theme 2: access to training and specialisation

Overall, clinicians spoke positively regarding the quality of training and clinical consultation provided by the OCD team. The 2-day workshops were often a highlight for clinicians, permitting dedicated time to upskill and learn from experts in the field, increasing their competency and confidence (Racz et al., 2025), and many reported enjoyment and professional fulfilment. The workshops increased evidence-based knowledge (Racz et al., 2025) but clinicians desired more practical skills training. Services were eager for further training and reported that more training – both in terms of depth of training and expansion to more staff – would be an enabler for implementation. Staff reported an increased awareness of OCD and subsequently noted increased detection as well as a greater likelihood of exploring possible OCD symptoms. They appreciated referring clients for specialised assessments, enhancing their conceptualisation and consulting with the speciality service about treatment directions or hurdles. About half of the managers and clinicians reported that they did not previously see the need for OCD-specific services but now appreciated their value.

The intensive ERP program (delivered by the OCD team using the OCD Busters program [Farrell et al., 2022]) was almost universally seen as a benefit to services, providing positive outcomes for clients who were making inadequate progress with standard therapy and a good fit for complex CAMHS clients. Clinicians valued opportunities for observation and/or co-facilitation of sessions to build their own ERP skills. Clinicians expressed a desire to learn to deliver the intensive program themselves rather than referring out to another service, although they acknowledged that current funding structures are not suited to the intensive structure (i.e., 3-hour sessions multiple times per week). Participants also reported difficulties with a lack of knowledge about referral processes and logistical concerns such as clients missing school to attend the program or scheduling clashes with pre-existing treatment sessions. Supervision was also almost universally well received, with clinicians commenting on its quality, the supervisor’s extensive experience and the practical approach to upskilling.

Services provided mixed feedback on the group ERP program. Some participants commented that they felt insufficiently trained to facilitate the group, particularly without an accompanying clinician manual, and/or that there were not enough staff trained to make running the group viable. They noted that the 10-week program duration was long, resulting in a time cost to clinicians, detracting from other service provisions. Additionally, comorbid problems were not an active focus of treatment during the program, which could result in client drop-out or regression/stagnation in other clinical problems. Running additional family sessions outside of the group was beyond the capacity of all services, unless facilitated by the OCD team, and as a result parent sessions could be difficult to contain within the three allocated sessions. All services noted that it could be difficult to reach a “critical mass” of clients, which created pressure to “find” clients who may be less suitable for the group and/or where OCD was not their primary concern. In contrast, others commented that they felt equipped to deliver groups and received professional enjoyment from implementing something new. Most clinicians observed direct benefit to both clients and parents through psychoeducation, symptom reduction and peer support, although a few clinicians reported little measurable improvement in OCD symptoms.

### Theme 3: impact of risk and clinical complexity

Clinicians reported finding the MoC easier to implement with clients who had a primary diagnosis of OCD and/or who had less comorbidity, complexity or risk. The wrap-around care and intensive treatment options were appropriate for the complex cases seen in CAMHS services, particularly for severe OCD. Most barriers related to screening and assessment, particularly where OCD was not the primary presentation. Three services that had not previously screened routinely for other conditions reported concerns regarding screening solely for OCD in initial assessments. This resulted in reduced screening, particularly if a high degree of risk or complexity (e.g., suicidality) took precedence or where queries relating to personality factors or demand characteristics impacted how a client might respond to screening (i.e., clients thinking the reason for screening relates to their presentation specifically). Clinicians queried the high false‑positive rate and low specificity of the selected screening measure, which had not been previously validated in routine public health settings (Dyason, Pham, et al., 2025), and reported concerns that clients may identify with, or find meaning in, a query of OCD prior to completing a full assessment. Respondents reported that screening could be burdensome on clients and services when OCD is not present and perceived utility is low. Clinicians preferred using clinical judgement to determine when, whether and how (e.g., verbally vs self-report) to screen for each client. Similar concerns were raised about diagnostic assessments. Additionally, some clinicians held concerns around differential diagnosis and found differences in young person and parent reports hard to integrate.

## Discussion

Prior to the current implementation trial, there were no public paediatric OCD-specialty services or OCD-specific treatment guidelines in Australia. Aligned with the UK’s NICE guidelines for OCD, research findings, clinical expertise and consultation with clinicians and consumers, we developed a tiered wraparound MoC for paediatric OCD, which was trialled across six child and adolescent mental health services. Overall, implementation was feasible with supports and tailoring to each service context. Some aspects of the model were more acceptable than others and to some services more than others. Although the MoC was adopted across the LHD, services had varied implementation and this was reflected in only five of six services participating in the formal qualitative interviews. Barriers and enablers to implementation tended to be directly and inversely related and fell into three broad categories: the pre-existing structures, supports and resources within services prior to commencing the implementation trial; access to training, supervision and specialised assessment/treatment through the OCD service; and the impact of risk or clinical complexity on implementing aspects of the MoC.

### Operational and technical feasibility

All six services successfully implemented at least some aspects of the MoC, with the training workshops in particular being attended by all six services. Feasibility was strongly linked to acceptability, where the most acceptable aspects to services were the easiest to implement. Services perceived adding structures to be easier to implement than changing existing procedures, which necessitated whole team collaboration.

The two least feasible aspects of the MoC were routine screening and the group ERP program. Four services did not already have structures in place to screen all clients at intake, so they needed to invent and develop de novo processes, resulting in three services not implementing screening in any form. The group ERP program was logistically challenging for services to implement, requiring room space, clinician scheduling and sometimes travel between sites. Additionally, groups required enough suitable and interested clients at similar stages in their treatment, which is challenging in CAMHS settings where clients are typically in active treatment, rather than being waitlisted or monitored. In contrast, headspace services have a larger throughput of clients in early stages of illness, and waitlisting is more common practice, meaning clients could be waitlisted for a few weeks until a group was running without significant change to processes.

Feasibility was bolstered by implementation support and training provided by the OCD team, where the OCD team could work with each service to tailor the MoC to each context or provide ongoing training and guidance on specific aspects, such as how to complete diagnostic assessments or think through differential diagnostics. Support from executives and health leadership was also invaluable in advocating for changing procedures and for safeguarding time for training and MoC clinical services, thereby overcoming some feasibility barriers.

Overall, certain aspects of the MoC “fit” better within some services than with others. Teams treating clients with moderate-severe mental health conditions (CAMHS, early psychosis team) were more invested in specialty training and tended to have lower staff turnover, so training represented better value for both the service and MoC team. Conversely, with more complex mental health problems, other clinical complexities or risks needed to be prioritised over perfect implementation procedures. Headspace had a higher throughput of clients to deliver treatment groups and already had routine screening procedures established for a wide variety of conditions. Two services experienced a mismatch between the funded elements of the trial for 7–17 years and their service population of 12–25 years, highlighting the discrepancy between “paediatric” and “youth” programs and measures. Youth services provide a bridge for young people from paediatric services to adult services, recognising both the peak onset and burden of mental health concerns occurring in this population, and the focus in adult mental health services on severe, relapsing and disabling mental illness, which is often inappropriate for emerging or milder mental health concerns where life transitions and family supports are still core focuses of treatment (McGorry, 2007). In contrast, research fields are typically divided into paediatric (under 18 years) and adult (over 18 years). In the current MoC implementation, the screening measure and group and intensive ERP programs were all empirically validated for 7–17 years, and the children’s hospital where the OCD team was based sees patients up to 18 years only. Reconciling the differences between paediatric and youth services was beyond the scope of the current pilot implementation trial, but should the MoC be implemented in the 18–25 population, both programs and measures will need to be adapted and re-validated for the unique needs of young adults.

### Acceptability

The intensive program and the training and supervision were highly acceptable and had uptake across all six services. Clinicians, in particular, reported professional satisfaction from building their knowledge and skills through expert training and supervision and from working alongside the specialist team to co-deliver care for complex clients, including through the intensive program. However, there remained a desire to treat clients within existing services, and clinicians reported wanting to upskill to deliver the intensive program themselves rather than referring outside their service.

The screening tool specifically had low acceptability. The selected screening tool had not been administered as a standalone screening tool in routine care (Dyason, Pham, et al., 2025), so the implementation trial assessed both the psychometric properties of the scale and its acceptability to the service. Unfortunately, the screener had low specificity and face validity in this setting (Dyason, Pham, et al., 2025), which was frequently noted as unacceptable to services, with flow-on effects across other aspects of the MoC such as referring positive screens for comprehensive diagnostic assessments. Using a different screening tool may have been more acceptable with improved implementation outcomes.

Clinicians suggested that being able to personalise assessments and treatments based on their clinical judgement and considering each client’s comorbidities, risk and treatment journeys – adapting both when and how various components should be implemented – would increase acceptability. However, diagnostic assessments are not typically a core remit of CAMHS or headspace; the most common principal diagnosis in CAMHS nationwide is recorded as “mental disorder not otherwise specified” (Brazel et al., 2023) so capacity may still need to be bolstered here before permitting more flexibility.

### Limitations

This pilot implementation trial provided valuable information to refine the MoC before larger-scale implementation. Nonetheless, some limitations are worth noting. First, although comments were live transcribed in front of interviewees, they were not recorded verbatim, prohibiting more advanced qualitative analysis. Second, while all members of the teams were invited to participate in the qualitative interviews, only five of the six services participated, and typically a small number of staff attended the interviews, representing the views of those who could not attend. This means different perspectives may not have been included in the logic model, particularly those of administrators or executive leadership who were often not present at the interviews and did not access training, yet were integral to successful implementation. There may also be a reciprocal link between how much services were willing/able to implement the MoC and how much time they allocated for staff to implement the MoC and subsequently attend qualitative interviews, potentially biasing results. Finally, interviews and thematic analyses were conducted by the OCD team. Interviewees may have tempered their feedback to maintain relationships with the OCD team, and/or the OCD team may have imposed their own beliefs and experiences of implementation onto the coding of themes or interpretation of results. In a larger-scale implementation trial, independent evaluators should conduct the interviews with full teams and executives, recording and transcribing comments to individual interviewees, and a separate team should then code the interviews to reduce bias.

### Conclusions and future directions

The MoC described here is the first MoC designed for paediatric OCD and implemented within an Australian public health context, based broadly on the UK’s pre-existing NICE guidelines (2005a) but also informed by knowledge of the Australian healthcare system, recent advances in the research literature and input from local services and consumers. The six participating services were located in one local health district but represented a spectrum of clinical severity and ranged from general, primary services to specialised tertiary services.

As a result of the implementation pilot, services reported significantly greater awareness of OCD as a condition and the need for specialised treatment and service plans. As such, the core working group still meets monthly and services continue to implement aspects of the MoC since the trial’s conclusion, expressing a desire for more training and increased resourcing to expand the OCD-specific services they offer. However, sustained adoption of procedures has been more challenging. Moving forward from the ideals of implementation to adoption and sustained change, more work is needed to overcome “short-termism” (Rapport et al., 2018). Implementation can often result in small changes that are hard to maintain, but behaviour change theories and implementation science frameworks can be leveraged to increase the likelihood of change (Presseau et al., 2022). More personnel resourcing within services and the implementation team could not only provide on-the-ground practical support to services but also engage across stakeholder groups, increase consultation with patients or lived‑experience consumers for co-design, engage with policymakers and remind implementors of the shared transformative goals (Rapport et al., 2018): better detection and treatment of OCD to relieve distress and suffering.

More stakeholder engagement could identify whose behaviours and which behaviours are vital to change at an organisational level and where fidelity is most important (Presseau et al., 2022) – for example, determining whether it is more effective or efficient to improve clinicians’ skills in detecting OCD or to work with team leaders to ensure consistent screening procedures are followed and positive screens receive comprehensive assessments. Economic modelling could dismantle which components are the most cost-effective for services, and whether increased costs in the short term save time and money in the long term. The work in this trial was funded through research grants and philanthropy; ensuring sustained implementation within public health services will require permanent, state-based funding. Finally, there also needs to be ongoing bidirectional mechanisms for iterative feedback and refinement of the MoC (Rapport et al., 2018): that the MoC can be adapted over time to fit within services and to each individual client, but equally ensuring there is an ability to correct course and improve fidelity to evidence-based care.

Given the well-documented and prominent “treatment and implementation quality gaps” (Farrell et al., 2023; Senter et al., 2021), there is a desperate need for a co-ordinated national approach to the detection and management of paediatric OCD that can be localised to unique service contexts to ensure successful and fit-for-purpose implementation. To enable this, substantial investment must occur within the health service – both financially and in terms of time – alongside strengthened collaboration and advocacy between services, clinicians, researchers, clients and families (Dyason et al., 2022). The pilot MoC described here offers a promising approach that, with refinement, increased adaptations to different service contexts, and expanded treatment options, could be rolled out on a larger scale across the state and then nationally.

## Supplementary Material

Clinician perspectives OCD MoC Supplemental Material

## Data Availability

The authors confirm that the data supporting the findings of this study are available within the article (Tables 1 and 2).
