# Supplementary material for: Implementing evidence-based care for paediatric obsessive-compulsive disorder in public mental health services: clinician perspectives of barriers and enablers
Source: Aust J Psychol. 2026 Jul 29;78(1):2692599. doi: 10.1080/00049530.2026.2692599 (PMC13421133; doi:10.1080/00049530.2026.2692599)
Supplement: Clinician perspectives OCD MoC Supplemental Material [file RAUP_A_2692599_SM3008.docx]

**OCD BOUNCE Qualitative Interviews August 2023**

| Interview Number |  | Team |  | Date |  | Time |  |
| --- | --- | --- | --- | --- | --- | --- | --- |
| Number of Team Members Present: | | | Clinicians trained in OCD Busters |  | Clinicians not trained | |  |
|  |  |  | Managers/team leaders trained |  | Managers/team leaders not trained | |  |
|  |  |  | Administrative staff |  |  |  |  |

Question Stems:

Initial question: In relation to *[Model of Care element]*, what *[Resources/Moderators/Mediators]* were necessary?

For example, *[examples listed]*. *Completed Stem Example: In relation to screening, what resources were necessary? For example, physical resources, staffing resources, pre-existing skills/training of staff prior to the Model being implemented.*

Follow-up questions: Was that [factor that was mentioned] a barrier or facilitator to implementing the Model of Care effectively? Was [that element] a good fit for your existing team processes?

|  | **Model of Care Elements (Activities)** | | | | | | |
| --- | --- | --- | --- | --- | --- | --- | --- |
|  | **Screening** | **Assessment** | **Group** | **Intensive** | **Training and Supervision** | **Monthly CPD** | **Integration of Model** |
| **Resources**  e.g., physical, staffing, pre-existing skills/training etc |  |  |  |  |  |  |  |
| **Moderators: Inner Factors**  e.g., organisational support, policy fit, resources, service pressures, autonomy, trust |  |  |  |  |  |  |  |
| **Moderators: Outer factors**  e.g., multidisciplinary team, leadership, relationships within and between teams, culture, staff wellbeing, staff experience/skills/interest, responsiveness to issues |  |  |  |  |  |  |  |
| **Mediators: Short-term outcomes**  e.g., established systems, shared agenda/ collaboration, implementation, morale, training, consumer/carer involvement, new connections, organisational change, client experience |  |  |  |  |  |  |  |
| **Mediators: Long-term outcomes**  e.g., embedded Model of Care, sustained improvements to client care |  |  |  |  |  |  |  |
